# Leukocytosis of Unknown Origin: Gangrenous Cholecystitis

**DOI:** 10.1155/2013/418014

**Published:** 2013-04-01

**Authors:** Amara Jyothi Nidimusili, M. Chadi Alraies, Naseem Eisa, Abdul Hamid Alraiyes, Khaldoon Shaheen

**Affiliations:** ^1^Department of Medicine, Trinitas Regional Medical Center, Seton Hall University Health Sciences, Elizabeth, NJ 07202, USA; ^2^Department of Hospital Medicine, Institute of Medicine, Cleveland Clinic Lerner College of Medicine of Case Western Reserve University, Cleveland Clinic, Cleveland, OH 44195, USA; ^3^Department of Hospital Medicine, Institute of Medicine, Cleveland Clinic, Cleveland, OH 44195, USA; ^4^Department of Pulmonary, Critical Care and Environmental Medicine, Tulane University Health Sciences Center, New Orleans, LA 70112, USA

## Abstract

There have been case reports where patients admitted with acute cholecystitis, who were managed conservatively, had subsequently developed GC (gangrenous cholecystitis). The current case is unique, since our patient denied any prior episodes of abdominal pain and the only tip off was leukocytosis. A high index of suspicion is essential for the early diagnosis and treatment of GC. GC has a mortality rate of up to 22% and a complication rate of 16–25%. Complications associated with GC include perforation, which has been reported to occur in as many as 10% of cases of acute cholecystitis. The radiological investigations may not be conclusive. Ultrasonography usually serves as the first-line imaging modality for the evaluation of patients with clinically suspected acute cholecystitis. However, CT can play an important role in the evaluation of these patients if sonography is inconclusive. There is a need for an early (if not urgent) surgical intervention in acute cholecystitis (whether laparoscopic or open surgery) in order to decrease the time elapsed from the start of symptoms to admission and treatment.

## 1. Introduction 

Gangrenous cholecystitis is one of the most severe forms of gallbladder inflammation, and accounts for minority of all patients with acute cholecystitis. It is the result of marked distension of the gallbladder causing increased tension in the gallbladder wall. Associated inflammation leads to ischemic necrosis of the wall. We have an atypical and unique presentation of a patient with painless gangrenous cholecystitis.

## 2. The Case

**Figure 1:**
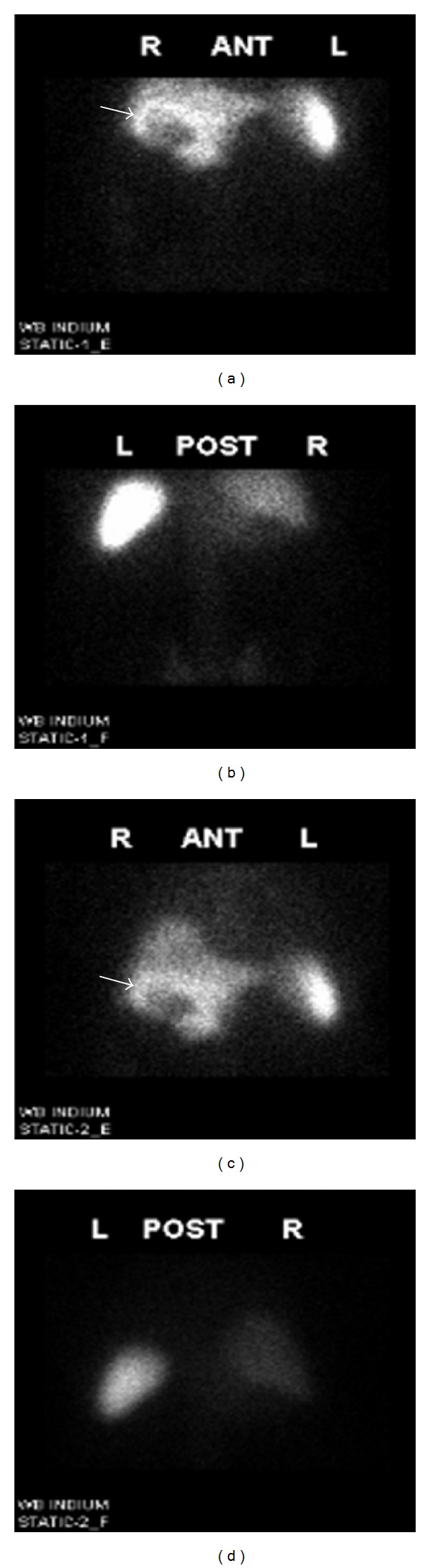
Twenty-four hours Indium-111 WBC gallbladder scan reveals increased accumulation of 111 in-leukocytes within the gallbladder wall suggestive of hyperemia/inflammatory changes in the pericholecystic region (arrow) (donut sign).

**Figure 2:**
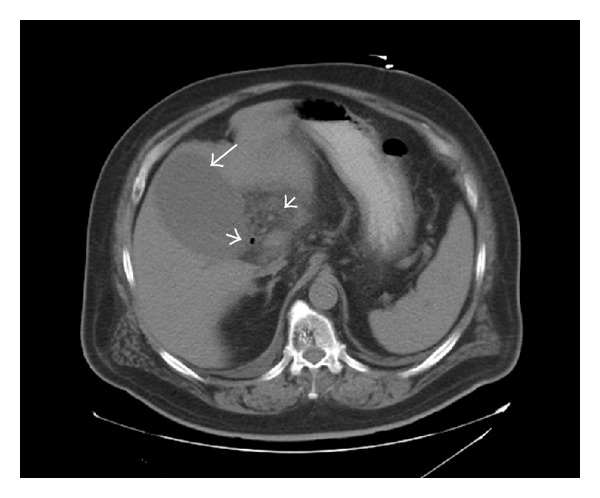
Computed tomography of the abdomen with oral contrast showing the gallbladder markedly distended (long arrow). Air bubbles project over the region of gallbladder neck are seen, suggestive of infection with gas-producing organism (short arrows).

A 66-year-old obese man with history of diabetes mellitus and hypertension was admitted to the hospital for atypical left-sided chest pain of several hours duration worsened by movement and relieved with nitroglycerin. Cardiac workup was negative and chest pain subsequently resolved. On presentation, he denied any abdominal pain, nausea, vomiting, fever, or chills. Physical examination was notable for heart rate of 110/minute, but other systemic examination was unremarkable. Initial workup showed WBC of 17,400/mm^3^ with 13% bands; aspartate aminotransferase (AST) 6 u/L; alanine aminotransferase (ALT) 23 u/L; alkaline phosphatase 80 u/L, total bilirubin was 0.9 mg/dL, and gamma-glutamyl transpeptidase (GGT) 55 U/L. During the hospital stay, leukocytosis persisted, with no possible source of infection had been identified. On day 3, blood cultures came back positive for methicillin-resistant *Staphylococcus epidermidis* (MRSE) and *Enterobacter sakazaki*. He was started on IV vancomycin and meropenem. Initially, transthoracic echocardiogram was negative with no evidence of vegetations. Abdominal ultrasound was done which showed multiple gallstones with normal sized gallbladder, with no wall thickening or pericholecystic fluid. Hepatobiliary iminodiacetic acid (HIDA) scan was inconclusive. Three days later, further testing to identify the cause of leukocytosis and source of infection included Indium WBC whole-body scan which showed increased accumulation of 111 in-leukocytes within the gallbladder wall suggestive of hyperemia/inflammatory changes in the pericholecystic region (Figure 1). Finally, CT scan of the abdomen showed a distended gallbladder with air bubbles project at the region of the gallbladder neck suggestive of an infection with gas-producing organisms and complicated cholecystitis (Figure 2). He had a laparoscopic surgery, where the gallbladder was found to be gangrenous and the procedure was advanced into an open cholecystectomy. Postsurgical hospital course was uneventful. He continued on antibiotics for additional two weeks and leukocytosis resolved.

## 3. Discussion

Gangrenous cholecystitis (GC) is an ominous progression of acute cholecystitis in which infection, inflammation, edema, bile stasis, and ischemia lead to gallbladder necrosis and perforation. The incidence of GC ranges from 2% to 29.6% in all patients with acute cholecystitis and generally occurs in older and diabetic patients [1]. 

Acute gangrenous cholecystitis is a medical and surgical emergency [1]. Some studies showed that the delay of hospitalization plays a crucial role in the formation of GC [2]. The time between the onset of symptoms and hospital admission was significantly longer in patients with GC. Furthermore, the patient's history (early or delayed admission) and physician's approach (general practitioner versus surgeon) are likely to play a role in the progression towards a severe necrosis of the gallbladder wall. GC has a mortality rate of up to 22% and a complication rate of 16–25%. A high index of suspicion is essential for the early diagnosis and treatment of GC [3]. Important prognostic factors that are associated with the development of acute gangrenous cholecystitis are as follows: age > 51 yrs, leukocyte count > 15,000, diabetes, African-American race, elevated ALT, AST, ALP, lipase levels, and pericholecystic fluid [1]. 

If the clinical diagnosis is cholecystitis, initial imaging is usually ultrasound (US). Sonographic signs of acute nongangrenous cholecystitis include intraluminal stones, impacted stone in the neck of gallbladder, and gallbladder tenderness (sonographic Murphy's sign). Gallbladder distension and wall thickening are nonspecific. In addition, the gallbladder wall thickening is often very marked and there may be a striated appearance to the wall or irregular protrusions within the lumen [4]. US is currently considered the preferred initial imaging technique for patient who is clinically suspected of having acute cholecystitis because of lower costs and availability after hours. However, the diagnostic accuracy of US has a substantial margin of error (sensitivity 81%) compared to other diagnostic modalities (Sensitivity of cholescintigraphy 96% and CT scan 94%). Therefore, US should be used to confirm the presence of gallbladder stones rather than to diagnose acute cholecystitis. Diagnostic accuracy of MR imaging is comparable to that of US [5]. 

On the other hand, in GC the sonographic Murphy's sign may be paradoxically absent, presumably because of necrosis and denervation of the gallbladder; its absence in a patient otherwise highly suspicious for acute cholecystitis raises the suspicion for GC. Emphysematous cholecystitis may exist without gangrene of the gallbladder or infection with gas-forming organisms. 

The accuracy of preoperative US in diagnosing GC remains uncertain. Twenty-eight percent of patients with GC had ultrasound reports that failed to show any evidence of acute inflammation as in our patient. This is mainly due to the absence of sonographic Murphy's sign and gallbladder walls of less than 3 mm, both important radiological signs of acute inflammation of the gallbladder [6]. CT scan is more useful in diagnosing acute cholecystitis. However, CT scan may fail to detect gallstones because many stones are isodense with bile. Furthermore, CT scan can be useful when complications of cholecystitis (such as emphysematous cholecystitis, gangrenous cholecystitis, or gallbladder perforation) are suspected or other diagnoses are considered [7]. The CT findings most specific for acute GC are gas in the wall or lumen, intraluminal membranes and irregular wall and pericholecystic abscess. GC is associated with a lack of mural enhancement, pericholecystic fluid, and a greater degree of gallbladder distension and wall thickening [4]. 

Tagged white blood cell scan uses autologous WBCs labeled with indium-111 oxyquinoline or gallium-67 [8]. The tagged white cells are given via intravenous rout. 24 hours later, images are taken. In general, those diagnostic modalities are highly sensitive and particularly efficacious of including the whole body. However, they cannot pinpoint a diagnosis; as a result, nonspecific tests localize a site for more specific evaluation such as with CT. When studied, indium-111 or gallium-67 labeled leukocyte scanning may be higher than with computed tomography (CT) or ultrasound, since the latter tests focus on only a few parts of the body. Therefore, Indium-111 or gallium-67 scanning is reserved for cases in which the initial evaluation remains negative and a screening evaluation at the entire body is desired [9]. In acute cholecystitis, the tagged white cells accumulate at the sites of inflammation or infection within the gallbladder wall (donut sign) [10] (Figure 1). In the uncommon situation where acalculous acute cholecystitis or incomplete cystic duct obstruction with cholecystitis is clinically suspected despite HIDA scan visualization, tagged WBCs may also be of value by demonstrating the inflammatory changes occurring within the gallbladder wall, although the cystic duct is patent.

The corner stone treatment for GC is surgery. In the hands of an experienced laparoscopic surgeon, an initial attempt at laparoscopic cholecystectomy is possible, converting to open procedure if necessary. The conversion rates range from 8% to 75% [11].

## 4. Conclusion

Our case emphasizes the importance of early detection of gangrenous cholecystitis when dealing with patients with acute cholecystitis. It is important to keep a high index of suspicion for the diagnosis of this condition in order to avoid potentially serious complications. Preoperative diagnosis of this condition may prove difficult.
